# Activation and activities of the p53 tumour suppressor protein

**DOI:** 10.1054/bjoc.2001.2128

**Published:** 2001-12

**Authors:** É Bálint, K H Vousden

**Affiliations:** NCI at Frederick, Building 560, Room 22–96, 1050 Boyles Street, Frederick, MD, 21702-1201, USA

## Abstract

The p53 tumour suppressor protein inhibits malignant progression by mediating cell cycle arrest, apoptosis or repair following cellular stress. One of the major regulators of p53 function is the MDM2 protein, and multiple forms of cellular stress activate p53 by inhibiting the MDM2-mediated degradation of p53. Mutations in p53, or disruption of the pathways that allow activation of p53, seem to be a general feature of all cancers. Here we review recent advances in our understanding of the pathways that regulate p53 and the pathways that are induced by p53, as well as their implications for cancer therapy. © 2001 Cancer Research Campaign http://www.bjcancer.com


*British Journal of Cancer* (2001) **85**(12), 1813–1823
© 2001 Cancer Research Campaign
doi: 10.1054/ bjoc.2001.2128, available online at http://www.idealibrary.com on IDEAL
http://www.bjcancer.com

### Activation and activities of the p53 tumour suppressor protein

**É Bálint and KH Vousden**

NCI at Frederick, Building 560, Room 22–96, 1050 Boyles Street, Frederick, MD 21702-1201, USA

**Summary** The p53 tumour suppressor protein inhibits malignant progression by mediating cell cycle arrest, apoptosis or repair following cellular stress. One of the major regulators of p53 function is the MDM2 protein, and multiple forms of cellular stress activate p53 by inhibiting the MDM2-mediated degradation of p53. Mutations in p53, or disruption of the pathways that allow activation of p53, seem to be a general feature of all cancers. Here we review recent advances in our understanding of the pathways that regulate p53 and the pathways that are induced by p53, as well as their implications for cancer therapy. © 2001 Cancer Research Campaign http://www.bjcancer.com

The *p53* gene is frequently mutated in sporadic cancer (Hainaut and Hollstein, 2000) and germline mutations in p53 result in Li–Fraumeni syndrome, a hereditary cancer susceptibility syndrome predisposing individuals to sarcomas, lymphomas, breast, brain and other tumours (Malkin et al, 1990; Srivistava et al, 1990). These findings are paralleled by the observation that mice deficient for p53 are highly tumour susceptible, although p53 deficiency does not prevent normal development (Donehower, 1996). Malignancies that retain the wild-type *p53* gene have often acquired other mechanisms to compromise p53 function, and most cancer cells show a defect either in p53 or in the pathway that leads to activation of p53 in response to oncogenic stimuli (Vogelstein et al, 2000). Taken together, inactivation of the p53 pathway seems to be a general mechanism in tumour development and might be a common feature of all cancers. Thus understanding the mechanisms that regulate p53 function has great importance for cancer therapy.

### CONTROL OF p53 FUNCTION

Since p53 is a powerful inhibitor of cell proliferation, control of its activity is essential during normal growth and development. Regulation of p53 has been described at the level of transcription, translation, conformational change, and various covalent and non-covalent modifications (Ashcroft and Vousden, 1999). However, at present it seems clear that one of the key mechanisms by which p53 function is regulated is through control of protein stability. Integral to this is the function of the MDM2 protein, and multiple forms of cellular stress activate p53 by countering the MDM2-mediated degradation of p53.

### Regulation by MDM2

MDM2 has been shown to inhibit p53 activity in several ways: by binding to the transactivation domain of p53, by targeting p53 for ubiquitination, by inhibiting acetylation of p53 and by shuttling p53 to the cytoplasm. Since the *MDM2* gene is a transcriptional target of p53 (Barak et al, 1993), an autoregulatory feedback loop exists in which p53 activates expression of its own negative regulator (Wu et al, 1993). The importance of MDM2 in the regulation of p53 activity is illustrated by the observation that MDM2 deficiency causes early embryonic lethality in mice which is rescued in the p53-null background (Jones et al, 1995; Montes de Oca Luna et al, 1995), indicating that in the absence of MDM2, unrestrained p53 activity blocks normal growth and development. Conversely, amplification of MDM2 is associated with the development of tumours that retain wild-type p53 (Oliner et al, 1992), suggesting that overexpression of MDM2 prevents the normal p53-mediated response to oncogenic stress.

MDM2 binds to p53 within the amino terminus of p53, (Figure 1) directly blocking the interaction of p53 with transcriptional coactivators (Momand et al, 1992; Wadgaonkar and Collins, 1999) and so inhibiting the ability of p53 to activate transcription of target genes. However, a more comprehensive inhibition of p53 function is achieved by the ability of MDM2 to promote proteasome-mediated degradation of p53 (Haupt et al, 1997; Kubbutat et al, 1997). MDM2 contains a RING finger domain (Figure 1) and, like many RING finger proteins, it can function in vitro as a ubiquitin protein ligase (E3) (Joazeiro and Weissman, 2000). MDM2 targets both itself and p53 for ubiquitination, and RING finger domain mutations lead to the stabilization of both proteins (Honda et al, 1997, Fang et al, 2000). Interestingly, the transcriptional cofactor, p300, that plays a role in acetylating and activating p53, also participates in the degradation of p53 by MDM2, possibly by functioning as a platform to allow efficient p53/MDM2 interaction (Grossman et al, 1998).

Besides direct ubiquitination of p53, MDM2 also plays a role in regulating the subcellular localization of p53. MDM2's ubiquitin ligase activity contributes to the efficient nuclear export of p53 (Boyd et al, 2000; Geyer et al, 2000), which depends on the nuclear export sequence (NES) identified in the C-terminus of p53 (Stommel et al, 1999). It is possible that ubiquitination of p53 reveals the NES, possibly by driving p53 into a monomeric form (Stommel et al, 1999), allowing access to the nuclear export machinery. Treatment of cells with leptomycin B, a drug that blocks nuclear export, results in stabilization and nuclear accumulation of p53 (Freedman and Levine, 1998; Lain et al, 1999; Tao and Levine, 1999) and mutation of the NES in p53 reduces, but does not abolish, the ability of MDM2 to target p53 for degradation (Yu et al,

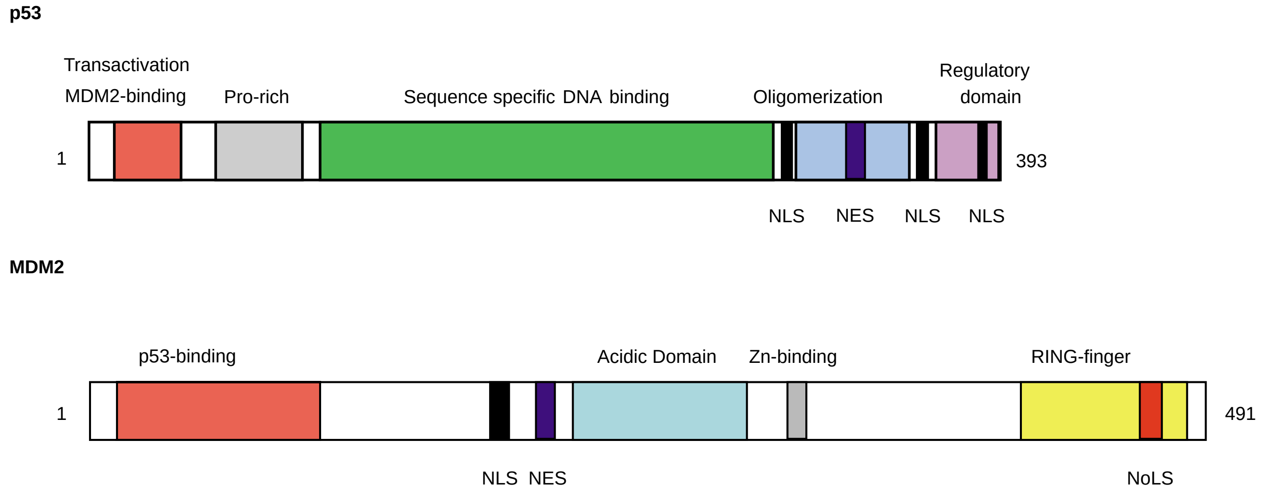

**Figure 1** Domain structure of the p53 and MDM2 proteins. NLS, nuclear localization signal; NES, nuclear export signal; NoLS, nucleolar localization signal

2000). Taken together, the results suggest that efficient degradation of p53 requires nuclear export, although some degradation can also occur in the nucleus, and indicate that ubiquitination of p53 by MDM2 may serve to both target p53 to the proteasome and allow export to the cytoplasm. In certain tumours, including neuroblastomas, breast and colon cancer, wild-type p53 is constitutively accumulated in the cytoplasm. Although this cytoplasmically sequestered p53 seems to be resistant to MDM2-mediated degradation because of post-translational modification (Zaika et al, 1999), recent evidence suggests that MDM2-mediated nuclear export might actually be responsible for the cytoplasmic accumulation of p53 (Lu et al, 2000). However, other studies indicate that in some tumours p53 may be sequestered in the cytoplasm through interaction with the glucocorticoid receptor (Sengupta et al, 2000).

In addition to its role in the regulation of p53, MDM2 has functions that are independent of p53 (Lundgren et al, 1997), with evidence that it can inhibit or promote cell growth depending on the cellular environment (Freedman et al, 1999). These activities of MDM2 are probably related to its interaction with other proteins, including regulators of the cell cycle and cell fate such as MTBP (Boyd et al, 2000), Numb (Juven-Gershon et al, 1998), the retinoblastoma tumour suppressor protein (pRB) (Hsieh et al, 1999), E2F1/DP1 (Loughran and La Thangue, 2000) and SMAD transcription factors (Yam et al, 1999). E2F-1 has been shown to be susceptible to proteasome-mediated degradation and, like p53, it can be stabilized by various forms of cellular stress (Blattner et al, 1999; O'Connor and Lu, 2000). However whether MDM2 participates in these processes remains unclear (Kubbutat et al, 1999; Loughran and La Thangue, 2000). MDM2 has been shown to bind ribosomal proteins and RNA (Marechal et al, 1994; Elenbaas et al, 1996), raising the question whether this contributes to the nucleolar localization of MDM2 seen in some circumstances (see below).

### MDM2-independent regulation of p53 stability

While MDM2 has been well established as the key regulator of p53 abundance and activity, various additional mechanisms have been described to regulate stability of p53. In cervical cancer cells, p53 is degraded due to ubiquitination by the human papillomavirus (HPV) E6 oncoprotein in complex with the cellular E6AP protein (Scheffner et al, 1993). Although these HPV-associated tumours usually retain a wild-type *p53* gene (Crook et al, 1992), the p53 response is severely compromised due to an inability to efficiently stabilize the p53 protein. However, E6AP does not appear to participate in the degradation of p53 in normal cells. Another cellular protein, JNK, was shown to bind p53 in normal, non-stressed cells. Interference with the p53-JNK association reduced p53 ubiquitination and increased p53 half-life, suggesting that JNK might target p53 to ubiquitination and degradation (Fuchs et al, 1998). In addition to degradation by the proteasome, p53 has been shown to be cleaved by another protease, calpain, which may also serve to regulate p53 (Kubbutat and Vousden, 1997; Pariat et al, 1997; Zhang et al, 1997; Atencio et al, 2000).

### Mechanisms to stabilize p53 in response to stress

### *1. Phosphorylation*

Many stress signals, including some DNA-damaging agents, modulate p53 and MDM2 activity through induction of kinases (Lakin and Jackson, 1999). Numerous phosphorylation sites within or near the N-terminal MDM2-binding region of p53 have been described (Meek, 1999; Ljungman, 2000) and phosphorylation at many of these sites can attenuate binding of p53 to MDM2 in vitro, potentially leading to stabilization of p53 in vivo (Unger et al, 1999; Chehab et al, 2000; Sakaguchi et al, 2000; Shieh et al, 1997, 2000). The kinases signalling to p53 include casein kinase 1 and 2, ATM (ataxia telangiectasia mutated), ATR (ATM/Rad3 related kinase) CHK1 and 2, JNK (jun N-terminal kinase) and DNA-PK (DNA-dependent protein kinase) (Jayaraman and Prives, 1999). Many of the same kinases also phosphorylate MDM2 in vitro, (Guerra et al, 1997; Mayo et al, 1997; Gotz et al, 1999; Khosravi et al, 1999) and MDM2 is heavily phosphorylated in vivo (Hay and Meek, 2000). Phosphorylation of MDM2 within the p53 binding domain, around the NLS, NES and in the acidic domain strongly suggests a regulatory role for these modifications (Hay and Meek, 2000; Maya and Oren, 2000).

Genetic evidence from both mice and humans suggests that ATM and CHK2 are key players in the pathway of response to ionizing radiation. The *ATM* gene is mutated in the genetic disorder ataxia telangiectasia, which is characterized by hypersensitivity to ionizing radiation and predisposition to cancer (Meyn, 1999), while Chk2 was found to be mutated in patients with Li–Fraumeni syndrome who do not carry mutations in p53 (Bell

 *© 2001 Cancer Research Campaign*

et al, 1999). Cells deficient for ATM or Chk2 show a defect in their ability to stabilize p53 following exposure to IR (Kastan et al, 1992; Hirao et al, 2000), strongly supporting a role for these kinases in this damage response.

### *2. Regulation of MDM2 expression*

Although phosphorylation probably plays an important role in some pathways leading to the stabilization of p53, other forms of stress can signal without a requirement for p53 phosphorylation (Ashcroft et al, 1999; Blattner et al, 1999). Several DNA-damaging agents including UV, topoisomerase inhibitors and non-genotoxic stress such as hypoxia, induce a p53 response by specific inhibition of MDM2 transcription (Wu and Levine, 1997; Arriola et al, 1999; Ashcroft et al, 2000; Ma et al, 2000; Zeng et al, 2000; Koumenis et al, 2001). Down-regulation of MDM2 was shown to be p53-independent, but the exact mechanisms are yet to be elucidated.

### *3. ARF*

The alternative reading frame product of the INK4A locus, called $p14^{ARF}$ (mouse $p19^{ARF}$) binds directly to MDM2, inhibiting the ubiquitin ligase activity of MDM2 and blocking the inhibition of p53 acetylation by MDM2 (Bates et al, 1998; Kamijo et al, 1998; Pomerantz et al, 1998; Stott et al, 1998; Zhang et al, 1998; Honda and Yasuda, 1999; Midgley et al, 2000; Ito et al, 2001). In some systems, ARF also leads to the relocalization of MDM2 from the nucleoplasm to the nucleolus (Weber et al, 1999) utilizing nucleolar localization signals in both ARF and MDM2 (Lohrum et al, 2000; Weber et al, 2000), and the consequence of ARF expression is the efficient stabilization and activation of p53. ARF plays an important role in the induction of p53 in response to oncogene activation, a critical fail-safe mechanism that eliminates cells with proliferative abnormalities. For example, deregulated E2F1 activity, which is seen in almost all cancers following disruption of the pRB tumour suppressor pathway, sends a strong apoptotic signal. Although part of this E2F1-induced apoptotic response is p53 independent (Phillips et al, 1999; Irwin et al, 2000), E2F1 transcriptionally activates ARF and the subsequent induction of p53 in response to deregulated E2F1 activity is an important component of the response. Similarly, the growth suppressive effects of activated Ras or Myc depend, in part, on the induction of p53 through ARF (Sherr and Weber, 2000). Other oncogenic events that induce a p53 response and might function through ARF include activation of the c-Abl protein-tyrosine kinase (Radfar et al, 1998; Sionov et al, 1999) and deregulation of beta-catenin, a frequent early event in colon carcinogenesis (Damalas et al, 1999). The importance of the ARF/p53 pathway is illustrated in mice, where deletion of ARF results in tumour development (Kamijo et al, 1997). Although mutations specific to ARF are rarely found in human tumours, loss of ARF expression resulting from methylation of the ARF promoter (Esteller, 2000), or overexpression of transcriptional repressors of ARF such as Twist (Maestro et al, 1999), Bmi-1 (Jacobs et al, 1999) or TBX2 (Jacobs et al, 2000) is associated with human cancer development.

### *4. Other regulators of p53 stability*

MDMX, a protein related to MDM2, also possesses a p53-binding domain and a RING finger domain. Binding of MDMX inhibits p53 transactivation function (Shvarts et al, 1996), although MDMX does not appear to target p53 for degradation. It is possible that MDMX does not show ubiquitin ligase activity, and hetero-oligomerization of MDMX with MDM2 through their RING finger domains results in the stabilization of MDM2 (Sharp et al, 1999; Tanimura et al, 1999). Furthermore, when overexpressed, MDMX protected p53 from MDM2-mediated degradation while still maintaining suppression of p53 transactivation (Jackson and Berberich, 2000). The role of MDMX in tumour development remains to be determined, although there is evidence that amplification of MDMX may substitute for p53 mutation or amplification of MDM2 in some tumours (Riemenschneider et al, 1999). Alternatively spliced forms of MDM2, lacking the N-terminal p53-binding domain, have also been described in human cancers and may play a role in regulating full-length MDM2 activity (Sigalas et al, 1996; Perry et al, 2000).

Several other proteins have been reported to interfere with the MDM2-mediated degradation of p53, although their mechanism of function is not well understood. pRB binds MDM2 and inhibits MDM2-dependent degradation of p53, with selective restoration of p53's apoptotic function (Hsieh et al, 1999). Other proteins that regulate p53 stability, and so potentially inhibit MDM2, include HIF1$\alpha$ (An et al, 1998) ING1 (Garkavtsev et al, 1998), WRN (Blander et al, 2000) and WT1 (Maheswaran et al, 1995).

### Regulation of p53 activity

In addition to the regulation of protein stability, there are mechanisms that regulate the activity of p53. The extreme C terminus of the protein controls its sequence-specific DNA binding and transcriptional activity, and these functions can be influenced by a multitude of covalent and non-covalent modifications within the C terminus. Modifications suggested to be involved in activation of p53 include sumoylation (Gostissa et al, 1999; Rodriguez et al, 1999; Muller et al, 2000), phosphorylation, dephosphorylation, acetylation, glycosylation (Shaw et al, 1996), ribosylation (Vaziri et al, 1997; Wang et al, 1998; Simbulan-Rosenthal et al, 1999) and redox regulation.

Transcriptional coactivators p300, CBP and PCAF have been shown to enhance p53-mediated transcription, and are important for p53 growth arrest and apoptotic functions. These coactivators bind to the N terminus and acetylate p53 at C-terminal lysine residues, thereby enhancing its sequence-specific DNA binding (Gu and Roeder, 1997). Phosphorylation of the N terminus of p53 enhances acetylation of the C terminus (Sakaguchi et al, 1998), and these modifications are DNA damage inducible (Sakaguchi et al, 1998; Liu et al, 1999). MDM2 can prevent this acetylation of p53 (Kobet et al, 2000; Ito et al, 2001), and association of p53 with deacetylating complexes provides further levels of control on p53 function (Juan et al, 2000; Luo et al, 2000). Acetylation of p53 is regulated by interaction with the promyelocytic leukaemia protein (PML), a RING domain containing tumour suppressor protein. Overexpression of PML relocalizes p53 into nuclear bodies and induces phosphorylation and acetylation of p53, thereby stimulating its transcriptional activity (Ferbeyre et al, 2000; Fogal et al, 2000; Guo et al, 2000; Pearson et al, 2000). Both DNA damage-induced apoptosis and oncogenic ras-induced senescence are impaired in PML-deficient cells, indicating an important role for PML in various p53-mediated stress responses.

Phosphorylation has also been shown to regulate p53 transcriptional activity. For example, the rapid phosphorylation on the C terminus on serine 392 in response to UV (Kapoor and Lozano, 1998) may stimulate sequence-specific DNA-binding activity of p53 (Hupp et al, 1992). A role for phosphorylation is also

*© 2001 Cancer Research Campaign* 

implicated in changing the oligomerization state of p53, or regulating its promoter specificity and the choice between apoptosis or cell cycle arrest (Lohrum and Scheidtmann, 1996; Sakaguchi et al, 1997; Oda et al, 2000). An ATM-dependent dephosphorylation of p53 at Ser376 has been described, creating a binding site for 14-3-3 proteins which, in turn, activate sequence-specific DNA binding of p53 (Waterman et al, 1998).

Non-covalent interaction with proteins such as Ref-1 and HMG-1 have also been shown to activate p53 DNA binding. Ref-1 is a multifunctional protein that participates in DNA repair through its abasic endonuclease activity and regulates the activity of several transcription factors by changing their redox state. Presumably by regulating the redox state of p53, it enhances trans-activation of p53 target promoters and increases p53-induced apoptosis (Jayaraman et al, 1997; Gaiddon et al, 1999). HMG-1 (high mobility group protein-1), a chromatin-associated non-histone protein, also increases the transcription of p53-dependent promoters, probably by inducing bending of the DNA (Jayaraman et al, 1998).

### THE p53 RESPONSE

The tumour suppressor function of p53 depends principally on its ability to prevent cellular proliferation in response to stress stimuli that are encountered during tumourigenic progression. Activated p53 leads to cell cycle arrest and apoptosis, and can play a role in the induction of differentiation and cellular senescence (Almog and Rotter, 1997; Lundberg et al, 2000). Wild-type p53 has been shown to inhibit angiogenesis in tumours, by activating or repressing genes that regulate new blood vessel formation (Dameron et al, 1994; Nishimori et al, 1997; Bouvet et al, 1998). p53 can also play a direct role in the repair of DNA damage, both through nucleotide excision repair and base excision repair (Ford and Hanawalt, 1995; Wani et al, 1999; Offer et al, 2001; Zhou et al, 2001).

### Cell cycle arrest

The cell cycle arrest function of p53 correlates well with its ability to function as a transcription factor (Crook et al, 1994; Pietenpol et al, 1994). Of the myriad of *p53* target genes identified to date $p21^{Waf1/Cip1}$ stands out as playing a critical role in the induction of cell cycle arrest (El-Deiry et al, 1993; Brugarolas et al, 1995). $p21^{Waf1/Cip1}$ is a cyclin-dependent kinase inhibitor that can activate both $G_1$ and $G_2$ cell cycle arrests similar to those seen in response to p53 induction (Harper et al, 1993; Agarwal et al, 1995; Bates et al, 1998). Importantly, cells deleted for $p21^{Waf1/Cip1}$ are deficient in both $G_1$ and $G_2$ arrest, and in the coupling of DNA synthesis and mitosis. These defects are also described in p53-null cells (Waldman et al, 1995; Waldman et al, 1996). Another target of p53 that contributes to the p53-induced $G_2$ arrest is 14-3-3 sigma (Hermeking et al, 1997). The 14-3-3 family proteins play a role in signal transduction and cell cycle control, in part by binding and sequestering proteins (Muslin and Xing, 2000). Although 14-3-3sigma deficient cells could transiently arrest in $G_2$ phase after DNA damage, they were unable to maintain the cell cycle arrest (Chan et al, 1999). As mentioned above, 14-3-3 can bind p53 and activate its sequence-specific DNA binding after IR (Waterman et al, 1998), and so may represent a positive feedback loop to p53 to prevent cell cycle progression in damaged cells. Further potential mediators of the G2 arrest include GADD45 (Wang et al, 1999) and Reprimo (Ohki et al, 2000).

### Apoptosis

While there is evidence that p53 can mediate apoptosis by transcription-independent mechanisms, p53 both activates and represses genes that participate in the apoptotic response. Cells in which the wild-type *p53* was replaced by a transcriptionally inactive mutant showed loss of both cell cycle arrest and apoptotic functions, supporting the importance of transcriptional regulation in these responses (Chao et al, 2000; Jimenez et al, 2000).

Numerous apoptotic genes that are transcriptionally activated by p53 have been identified, suggesting that the p53 apoptotic response is multifaceted (Vousden, 2000) (Figure 2). The first apoptotic target of p53 identified was the bax gene, a pro-apoptotic member of the BCL-2 family (Miyashita and Reed, 1995). Recently, other pro-apoptotic members of this family named Noxa (Oda et al, 2000) and PUMA (Nakano and Vousden, 2001; Yu et al, 2001) have been identified as p53 targets. These proteins, as well as another *p53* target gene product, p53AIP1 (Oda et al, 2000), localize to the mitochondria and promote loss of the mitochondrial membrane potential and cytochrome c release, thus activating the Apaf-1/caspase-9 apoptotic cascade (Bossy-Wetzel and Green, 1999). Significantly, p53-induced apoptosis was found to be inhibited by loss of Apaf-1 or caspase-9 (Soengas et al, 1999). Perturbation of mitochondrial integrity may also be mediated by several genes coding for redox-controlling enzymes, which were identified as *p53*-induced genes (PIGs) in a colon cell line undergoing p53-mediated apoptosis (Polyak et al, 1997). It has been proposed that reactive oxygen species (ROS) produced by these PIGs cause damage to mitochondria which in turn initiates apoptosis. This model is supported by the observations that antioxidants, which eliminate ROS, can inhibit p53-mediated apoptosis as well as concomitant changes in the mitochondrial membrane potential in some systems (Li et al, 1999). Recently a study revealed that the p53 protein itself can localize to the mitochondria presenting a potential additional transcription-independent way of mediating apoptosis (Marchenko et al, 2000).

p53 has also been implicated in the membrane death receptor-induced pathway of apoptosis in several ways. Expression of at least two of the death receptors, FAS/APO1 and DR5/KILLER, and one of the death receptor ligands, FASL, have been observed to be up-regulated by p53 (Owen-Schaub et al, 1995; Wu et al, 1997). The DR5 promoter was shown to be a direct target of p53 (Takimoto and El-Deiry, 2000), while cell surface expression of FAS was enhanced by p53 through promotion of its trafficking from the Golgi to the plasma membrane (Bennett et al, 1998). Activation of death receptors by their ligands (FAS by FASL and DR5 by TRAIL) results in trimerization and recruitment of intracellular adapter molecules which initiate the caspase cleavage cascade and apoptosis (Ashkenazi and Dixit, 1998). Activation of PIDD, a death domain containing protein, by p53 also induces apoptosis and is likely to function through the death receptor pathway (Lin et al, 2000).

Loss of survival signalling can augment p53-mediated apoptosis (Gottlieb et al, 1994; Abrahamson et al, 1995; Canman et al, 1995; Lin and Benchimol, 1995; Prisco et al, 1997), and the ability of p53 to negatively regulate the IGF pathway (Buckbinder et al, 1995) and inhibit intergrin-associated survival signalling may further sensitize cells to p53-induced death (Bachelder et al, 1999). The NF-κB transcription factor has lately been shown to play an important role in p53-mediated apoptosis (Ryan et al, 2000), in contrast to the anti-apoptotic effect of NF-κB induced in

 *© 2001 Cancer Research Campaign*

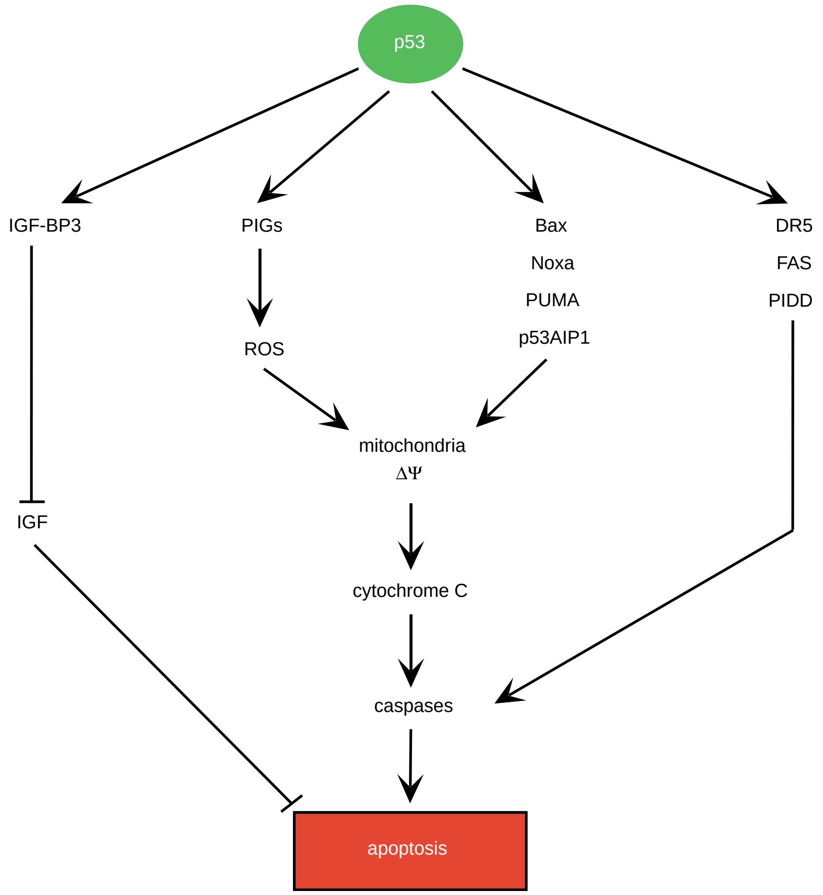

**Figure 2** p53 induces apoptosis by activating target genes such as PIGs, Bax, Noxa, PUMA, p53AIP1 DR5, FAS PIDD and IGF-BP3. p53-induced apoptosis involves change in mitochondrial membrane potential (ΔY), cytochrome C release and caspase activation response to TNF (Van Antwerp et al, 1996; Phillips et al, 1999). However, in other systems p53 expression has been shown to be dependent on NF-κB (Wu and Lozano, 1994; Kirch et al, 1999), and the contribution of NF-kB to the p53 apoptotic pathway remains unclear.

### Choice of response

Whether a cell undergoes cell cycle arrest or apoptosis in response to p53 depends on several factors. Some of these may be independent of p53, such as the presence of extracellular survival factors, the presence of other oncogenic alterations and the availability of additional transcription factors or cofactors (Vousden, 2000). However, the activity of p53 can also contribute to the choice of response. The type and the magnitude of the cellular stress may control p53 function by affecting the level or activity of the p53 protein that is induced. Activation of apoptosis has been associated with higher levels of p53 than those required for cell cycle arrest (Chen et al, 1996), suggesting that the promoters regulating expression of apoptotic genes bind p53 with a lower affinity than the cell cycle arrest targets. Alternatively, affinity of p53 to target promoters might be regulated by conformational change (Thornborrow and Manfredi, 1999), and several mutants of p53 show selective loss of the ability to activate apoptotic target genes and to induce apoptosis (Ryan and Vousden, 1998). Covalent modification such as phosphorylation can also regulate conformation and/or promoter specificity of p53. Phosphorylation of p53 on serine 46, for example, is required for the induction of the apoptotic target gene *p53AIP1* (Oda et al, 2000) and inhibition of the kinase responsible for serine 46 phosphorylation by the phosphatase WIP1, which is also p53-inducible, inhibits the ability of p53 to activate the apoptotic response (Takekawa et al, 2000).

### DNA repair

Besides preventing cells with damaged genomes from replicating, via its apoptotic and cell cycle arrest function, p53 also participates in DNA damage repair. Cells lacking p53 function are deficient in nucleotide excision repair (NER), which repairs UV-induced DNA damage (Ford and Hanawalt, 1995; Wani et al, 1999) and base excision repair (BER), which removes bases damaged by alkylating agents, oxygen-free radicals or hydrolysis (Offer et al, 2001; Zhou et al, 2001). The C-terminus of p53 directly binds to different forms of damaged DNA: single-stranded DNA, ends of double-strand breaks and DNA ‘bulges’ resulting from insertion/deletion mismatches. Also, p53 can associate with several components of the repair machinery in vitro, including XPB/ERCC3, XPD/ERCC2, p62 subunit of TFIIH, CSB, replication protein A and Ref-1. Other biochemical activities of p53, such as DNA reannealing, DNA strand transfer and 3′–5′ exonuclease activity might also play a role in its repair function (for review see Albrechtsen et al (1999) McKay et al (1999)).

Some of the *p53* target genes also participate in DNA damage repair. GADD45 binds proliferating cell nuclear antigen (PCNA),

© 2001 Cancer Research Campaign

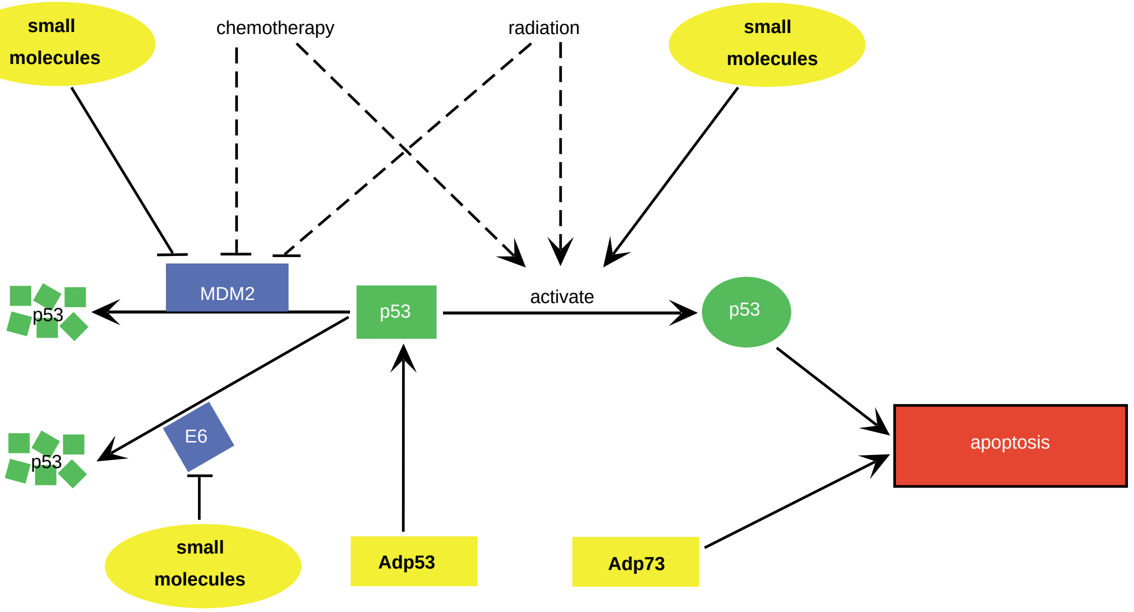

**Figure 3** In tumour cells with p53, radiation and certain chemotherapeutic agents activate p53 by various mechanisms discussed in the text. Small molecules can be used to inhibit MDM2- or E6-mediated degradation of p53 or to stabilize p53 in the active conformation. Alternatively, p53 or its homologues can be introduced by adenoviral vectors (Adp53, Adp73). Gene therapy is used in combination with radiation or chemotherapy and could inhibit replicative DNA synthesis, thus allowing DNA repair to proceed (Smith et al, 1994). Gadd45-null fibroblasts have defects in NER similar to those seen in p53-null fibroblasts (Smith et al, 2000) and Gadd45-deficient mice show increased radiation carcinogenesis and genomic instability comparable to that seen in p53-deficient mice (Hollander et al, 1999). Another transcriptional target of p53 that plays a role in DNA repair is p53R2, a ribonucleotide reductase gene (Nakano et al, 2000; Tanaka et al, 2000).

### POTENTIAL OF p53 IN CANCER THERAPY

Since most cancers are defective in the p53 response, and tumour cells are generally more sensitive to p53-mediated death than many normal cells, reintroduction or reactivation of p53 in tumour cells may have profound therapeutic utility. Several approaches to restore p53 function in tumour cells are presently being pursued (Figure 3). Mutant p53 can be re-activated in cells by small peptides derived from the C-terminus of p53 (Selivanova et al, 1997). Even more promising, small compounds were identified that stabilize both wild-type and mutant *p53* in the active conformation and thus are able to slow tumour growth in mice (Foster et al, 1999). Inhibition of MDM2 may be effective in tumours that retain wild-type *p53*, but fail to properly activate it, due to MDM2 overexpression or loss of ARF. Small peptides or antisense oligonucleotides that target MDM2 have been shown to activate p53 successfully in p53-positive tumour cells (Böttger et al, 1997; Chen et al, 1999). Similarly, inhibition of the HPV E6 protein can induce p53 in cervical cancer cells (Butz et al, 2000; Hietanes et al, 2000). These approaches may be less effective, however, in those cancers where resistance to p53-mediated tumour suppression results from defects in downstream effectors, such as Bax (Rampino et al, 1997) or Apaf-1 (Soengas et al, 2001).

Adenoviral or retroviral vectors have been used to re-introduce wild-type *p53* into tumour cells with no or mutant p53, inducing apoptosis and promoting tumour regression in combination with radiation therapy in clinical trials (Roth et al, 1999; Zeimet et al, 2000). Adenoviral expression of the p53 family members p63 or p73 was also able to induce apoptosis in certain cancer cells (Ishida et al, 2000), and since p73 is resistant to degradation by MDM2 and E6 (Marin et al, 1998; Prabhu et al, 1998; Balint et al, 1999; Dobbelstein et al, 1999; Ongkeko et al, 1999; Zeng et al, 1999), its potential for therapeutic application may be promising. In another approach, an adenovirus lacking the p53-inactivating oncogene E1B (Onyx-015) was shown to be able to replicate only in tumour cells that are defective in the p53 pathway, but not in normal cells, thus causing selective tumour cell killing and tumour regression in many patients (McCormick, 2000).

Despite the enhanced sensitivity of many cancer cells to p53-mediated death, many normal cells also undergo apoptosis in response to radiation or chemotherapy, leading to the debilitating side effects that limit the extent of chemotherapy that can be tolerated. In a contrasting approach, an inhibitor of p53 was shown to protect mice from the lethal effects of radiation treatment by preventing damage of wild-type *p53*-containing normal tissues (Komarov et al, 1999). Although many questions remain unanswered, it is apparent that our improving insights into the regulation and function of the p53 tumour suppressor will yield exciting advances in cancer therapy.

### REFERENCES

Abrahamson JLA, Lee JM and Bernstein A (1995) Regulation of p53-mediated apoptosis and cell cycle arrest by steel factor. *Mol Cell Biol* **15**: 6953–6960

Agarwal ML, Agarwal A, Taylor WR and Stark GR (1995) p53 controls both the G2/M and the G1 cell cycle checkpoints and mediates reversible growth arrest in human fibroblasts. *Proc Natl Acad Sci USA* **92**: 8493–9497

Albrechtsen N, Dornreiter I, Grosse F, Kim E, Wiesmuller L and Deppert W (1999) Maintenance of genomic integrity by p53: complementary roles for activated and non-activated p53. *Oncogene* **18**: 7706–7717

Almog N and Rotter V (1997) Involvement of p53 in cell differentiation and development. *Biochem Biophys Acta* **1333**: F1–27

An WG, Kanekal M, Simon MC, Maltepe E, Blagosklonny MV and Neckers LM (1998) Stabilization of wild-type by hypoxia-inducible factor 1α. *Nature* **392**: 405–408

 *© 2001 Cancer Research Campaign*

Arriola EL, Rodriguez Lopez A and Chresta CM (1999) Differential regulation of p21waf-1/cip-1 and Mdm2 by etoposide: etoposide inhibits the p53-Mdm2 autoregulatory loop. *Oncogene* **18**: 1081–1091

Ashcroft M and Vousden KH (1999) Regulation of p53 stability. *Oncogene* **18**: 7637–7643

Ashcroft M, Kubbutat MH and Vousden KH (1999) Regulation of p53 function and stability by phosphorylation. *Mol Cell Biol* **19**: 1751–1758

Ashcroft M, Taya Y and Vousden KH (2000) Stress signals utilize multiple pathways to stabilize p53. *Mol Cell Biol* **20**: 3224–3233

Ashkenazi A and Dixit VM (1998) Death receptors: Signaling and modulation. *Science* **281**: 1305–1308

Atencio IA, Ramachandra M, Shabram P and Demers GW (2000) Calpain inhibitor 1 activates p53-dependent apoptosis in tumor cell lines. *Cell Growth Differ* **11**: 247–253

Bachelder RE, Ribick MJ, Marchetti A, Falcioni R, Soddu S, Davis KR and Mercurio AM (1999) p53 inhibits alpha 6 beta 4 integrin signaling by promoting the caspase 3-dependent cleavage of AKT/PKB. *J Cell Biol* **147**: 1063–1072

Balint E, Bates S and Vousden KH (1999) Mdm2 binds p73α without targeting degradation. *Oncogene* **18**: 3923–3929

Barak Y, Juven T, Haffner R and Oren M (1993) Mdm-2 expression is induced by wild type p53 activity. *EMBO J* **12**: 461–468

Bates S, Phillips AC, Clarke PA, Stott F, Peters G, Ludwig RL and Vousden KH (1998a) p14ARF links the tumour suppressors RB and p53. *Nature* **395**: 124–125

Bates S, Ryan KM, Phillips AC and Vousden KH (1998b) Cell cycle arrest and DNA endoreduplication following p21Waf1/Cip1 expression. *Oncogene* **17**: 1691–1703

Bell DW, Varley JM, Szydlo TE, Kang DH, Wahrer DC, Shannon KE, Lubratovich M, Verselis SJ, Isselbacher KJ, Fraumeni JF, Birch JM, Li FP, Garber JE and Haber DA (1999) Heterozygous germ line hCHK2 mutations in Li-Fraumeni syndrome. *Science* **286**: 2528–2531

Bennett M, Macdonald K, Chan S-W, Luzio JP, Simari R and Weissberg (1998) Cell surface trafficking of Fas: a rapid mechanism of p53 mediated apoptosis. *Science* **282**: 290–293

Blander G, Zalle N, Leal JF, Bar-Or RL, Yu CE and Oren M (2000) The Werner syndrome protein contributes to induction of p53 by DNA damage. *FASEB J* **14**: 2138–2140

Blattner C, Tobiasch E, Litfen M, Rahmsdorf HJ and Herrlich P (1999) DNA damage induced p53 stabilization: no indication for an involvement of p53 phosphorylation. *Oncogene* **18**: 1723–1732

Bossy-Wetzel E and Green DR (1999) Apoptosis: checkpoint at the mitochondrial frontier. *Mutat Res* **434**: 243–251

Böttger A, Böttger V, Sparks A, W-L L, Howard SF and Lane DP (1997) Design of a synthetic Mdm-2 binding mini protein that activates the p53 response *in vivo*. *Current Biology* **7**: 860–869

Bouvet M, Ellis LM, Nishizaki M, Fujuwara T, Liu W, Bucana CD, Fang B, Lee JJ and Roth JA (1998) Adenovirus-mediated wild-type p53 gene transfer down-regulates vascular endothelial growth factor expression and inhibits angiogenesis in human colon cancer. *Cancer Res* **58**: 2288–2292

Boyd MT, Vlatkovic N and Haines DS (2000) A novel cellular protein (MTBP) binds to MDM2 and induces a G1 arrest that is suppressed by MDM2. *J Biol Chem* **275**: 31883–31890

Boyd SD, Tsai KY and Jacks T (2000) An intact HDM2 RING-finger domain is required for nuclear exclusion of p53. *Nature Cell Biol* **2**: 563–568

Brugarolas J, Chandrasekaran C, Gordon JI, Beach D, Jacks T and Hannon GJ (1995) Radiation-induced cell cycle arrest compromised by p21 deficiency. *Nature* **377**: 552–556

Buckbinder L, Talbott R, Velasco-Miguel S, Takenaka I, Faha B, Seizinger BR and Kley N (1995) Induction of the growth inhibitor IGF-binding protein 3 by p53. *Nature* **377**: 646–649

Butz K, Denk C, Ullmann A, Scheffner M and Hoppe-Seyler F (2000) Induction of apoptosis in human papillomavirus-positive cancer cells by peptide aptamers targeting the viral E6 oncoprotein. *Proc Natl Acad Sci USA* **12**: 6693–6697

Canman CE, Gilmer TM, Coutts SB and Kastan MB (1995) Growth factor modulation of p53-mediated growth arrest versus apoptosis. *Genes and Dev* **9**: 600–611

Chan TA, Hermeking H, Lengauer C, Kinzler KW and Vogelstein B (1999) 14-3-3 sigma is required to prevent mitotic catastrophe after DNA damage. *Nature* **401**: 616–620

Chao C, Saito S, Kang J, Anderson CW, Appella E and Xu Y (2000) p53 transcriptional activity is essential for p53-dependent apoptosis following DNA damage. *EMBO J* **19**: 4967–4975

Chehab NH, Malikzay A, Appel M and Halazonetis TD (2000) Chk2/hCds1 functions as a DNA damage checkpoint in G1 by stabilizing p53. *Genes and Dev* **14**: 278–288

Chen L, Lu W, Agrawal S, Zhou W, Zhang R and Chen J (1999) Ubiquitous induction of p53 in tumor cells by antisense inhibition of MDM2 expression. *Mol Med* **5**: 21–34

Chen X, Ko LJ, Jayaraman L and Prives C (1996) p53 levels, functional domains, and DNA damage determine the extent of the apoptotic response of tumor cells. *Genes and Dev* **10**: 2438–2451

Crook T, Wrede D, Tidy JA, Mason WP, Evans DJ and Vousden KH (1992) Clonal p53 mutation in primary cervical cancer: association with human-papillomavirus-negative tumours. *Lancet* **339**: 1070–1073

Crook T, Marston NJ, Sara EA and Vousden KH (1994) Transcriptional activation by p53 correlates with suppression of growth but not transformation. *Cell* **79**: 817–827

Damalas A, Ben-Ze’ev A, Simcha I, Shtutman M, Fernando J, Leal M, Zhurinsky J, Geiger B and Oren M (1999) Excess β-catenin promotes accumulation of transcriptionally active p53. *EMBO J* **18**: 3054–3063

Dameron KM, Volpert OV, Tainsky MA and Bouck N (1994) Control of angiogenesis in fibroblasts by p53 regulation of thrombospondin-1. *Science* **265**: 1582–1584

Dobbelstein M, Wienzek S, Koenig S and Roth J (1999) Inactivation of the p53-homologue p73 by the mdm2-oncoprotein. *Oncogene* **18**: 2101–2106

Donehower LA (1996) The p53-deficient mouse: a model for basic and applied cancer studies. *Sem Cancer Biol* **7**: 269–278

El-Deiry W, Tokino T, Velculescu VE, Levy DB, Parson VE, Trent JM, Lin D, Mercer WE, Kinzler KW and Vogelstein B (1993) WAF1, a potential mediator of p53 tumour suppression. *Cell* **75**: 817–825

Elenbaas B, Dobbelstein M, Roth J, Shenk T and Levine AJ (1996) The MDM2 oncoprotein binds specifically to RNA through its RING finger domain. *Mol Med* **2**: 439–451

Esteller M (2000) Epigenetic lesions causing genetic lesions in human cancer. Promoter hypermethylation of DNA reapir genes. *Eur J Cancer* **36**: 2294–2230

Fang S, Jensen JP, Ludwig RL, Vousden KH and Weissman AM (2000) Ubiquitin protein ligase activity of Mdm2: Differential RING finger requirements for ubiquitination and proteasomal targeting of Mdm2 and p53. *J Biol Chem* **275**: 8945–8951

Ferbeyre G, de Stanchina E, Querid E, Baptiste N, Prives C and Lowe SW (2000) PML is induced by oncogenic *ras* and promotes premature senescence. *Genes and Dev* **14**: 2015–2027

Fogal V, Gostissa M, Sandy P, Zacchi P, Sternsdorf T, Jensen K, Pandolfi PP, Will H, Schneider C and Del Sal G (2000) Regulation of p53 activity in nuclear bodies by a specific PML isoform. *EMBO J* **19**: 6185–6195

Ford JM and Hanawalt PC (1995) Li-Fraumeni syndrome fibroblasts homozygous for p53 mutations are deficient in global DNA repair but exhibit normal transcription-coupled repair and enhanced UV resistance. *Proc Natl Acad Sci USA* **92**: 8876–8880

Foster BA, Coffey HA, Morin MJ and Rastinejad F (1999) Pharmacological rescue of mutant p53 conformation and function. *Science* **286**: 2507–2510

Freedman DA and Levine AJ (1998) Nuclear export is required for degradation of endogenous p53 by MDM2 and human papillomavirus E6. *Mol Cell Biol* **18**: 7288–7293

Freedman DA, Wu L and Levine AJ (1999) Functions of the MDM2 oncoprotein. *Cell Mol Life Sci* **55**: 96–107

Fuchs SY, Adler V, Buschmann T, Yin Z, Wu X, Jones SN and Ronai Z (1998) JNK targets p53 ubiquitination and degradation in nonstressed cells. *Genes and Dev* **12**: 2658–2663

Gaiddon C, Moorthy NC and Prives C (1999) Ref-1 regulates the transactivation and pro-apoptotic functions of p53 in vivo. *EMBO J* **18**: 5609–5621

Garkavtsev I, Grigorian IA, Ossovskaya VS, Chernov MV, Chumakov PM and Gudkov AV (1998) The candidate tumour suppressor p33ING1 cooperates with p53 in cell growth control. *Nature* **391**: 295–298

Geyer RK, Yu ZK and Maki CG (2000) The MDM2 RING-finger domain is required to promote p53 nuclear export. *Nature Cell Biol* **2**: 569–573

Gostissa M, Hengstermann A, Fogal V, Sandy P, Scheffner M and Del Sal G (1999) Activation of p53 by conjugation to the ubiqutin-like protein SUMO-1. *EMBO J* **18**: 6462–6471

Gottlieb E, Haffner R, von Ruden T, Wagner EF and Oren M (1994) Down-regulation of wild-type p53 activity interferes with apoptosis of IL-3 dependent hematopoietic cells following IL-3 withdrawal. *EMBO J* **13**: 1368–1374

Gotz C, Kartarius S, Scholtes P, Nastainczyk W and Montenarh M (1999) Identification of a CK2 phosphorylation site in mdm2. *Eur J Biochem* **266**: 493–501

Grossman SR, Perez M, Kung AL, Joseph M, Mansur C, Xiao ZX, Kumar S, Howley PM and Livingston DM (1998) p300/MDM2 complexes participate in MDM2-mediated p53 degradation. *Mol Cell* **2**: 405–415

Gu W and Roeder RG (1997) Activation of p53 sequence-specific DNA binding by acetylation of the C-terminal domain. *Cell* **90**: 595–606

© 2001 Cancer Research Campaign 

Guerra B, Gotz C, Wagner P, Montenarh M and Issinger OG (1997) The carboxy terminus of p53 mimics the polylysine effect of protein kinase CK2-catalyzed MDM2 phosphorylation. *Oncogene* **14**: 2683–2688

Guo A, Salomoni P, Luo J, Shih A, Zhong S, Gu W and Pandolfi PP (2000) The function of PML on p53-dependent apoptosis. *Nature Cell Biol* **2**: 730–736

Hainaut P and Hollstein M (2000) p53 and human cancer; the first ten thousand mutations. *Adv Cancer Res* **77**: 81–137

Harper JW, Adami GR, Wei N, Keyomarsi K and Elledge SJ (1993) The p21 CDK-interacting protein cip1 is a potent inhibitor of G1 cyclin-dependent kinases. *Cell* **75**: 805–816

Haupt Y, Maya R, Kazaz A and Oren M (1997) Mdm2 promotes the rapid degradation of p53. *Nature* **387**: 296–299

Hay TJ and Meek DW (2000) Multiple sites of in vivo phosphorylation in the MDM2 oncoprotein cluster within two important functional domains. *FEBS Let* **478**: 183–186

Hermeking H, Lengauer C, Polyak K, He T-C, Zhang L, Thiagalingam S, Kinzler KW and Vogelstein B (1997) *14-3-3σ* is a p53-regulated inhibitor of G2/M progression. *Mol Cell* **1**: 3–11

Hietanes S, Lain S, Krausz E, Blattner C and Lane DP (2000) Activation of p53 in cervical carcinoma cells by small molecules. *Proc Natl Acad Sci USA* **97**: 8501–8506

Hirao A, Kong YY, Matsuoka S, Wakeham A, Ruland J, Yoshida HDL, Elledge SJ and Mak TW (2000) DNA damage-inducible activation of p53 by the checkpoint kinase Chk2. *Science* **287**: 1824–1827

Hollander MC, Sheikh MS, Bulavin DV, Lundgren K, Augeri-Henmueller L, Shehee R, Molinaro TA, Kim KE, Tolosa E, Ashwell JD, Rosenberg MP, Zhan Q, Fernandz-Salguero PM, Morgan WF, Deng CX and Fornace AJ (1999) Genomic instability in Gadd45a-deficient mice. *Nature Genet* **23**: 176–184

Honda R, Tanaka H and Yasuda H (1997) Oncoprotein MDM2 is a ubiquitin ligase E3 for tumor suppressor p53. *FEBS Lett* **420**: 25–27

Honda R and Yasuda H (1999) Association of p19$^{ARF}$ with Mdm2 inhibits ubiquitin ligase activity of Mdm2 for tumor suppressor p53. *EMBO J* **18**: 22–27

Hsieh JK, Chan FS, O'Connor DJ, Mittnacht S, Zhong S and Lu X (1999) RB regulates the stability and the apoptotic function of p53 via MDM2. *Mol Cell* **3**: 181–193

Hupp TR, Meek DW, Midgley CA and Lane DP (1992) Regulation of the specific DNA binding function of p53. *Cell* **71**: 875–886

Irwin M, Marin MC, Phillips AC, Seelan RS, Smith DI, Liu W, Flores ER, Tsai KY, Jacks T, Vousden KH and Kaelin WG (2000) Role for the p53 homolog p73 in E2F1-induced apoptosis. *Nature* **407**: 645–648

Ishida S, Yamashita T, Makaya U and Tokino T (2000) Adenovirus-mediated transfer of p53-related genes induces apoptosis of human cancer cells. *Jpn J Cancer Res* **91**: 174–180

Ito A, Lai CH, Zhao X, Saito S, Hamilton MH, Appella E and Yao T-P (2001) p300/CBP-mediated p53 acetylation is commonly induced by p53-activating agents and inhibited by MDM2. *EMBO J* **20**: 1331–1340

Jackson MW and Berberich SJ (2000) MdmX protects p53 from Mdm2-mediated degradation. *Mol Cell Biol* **20**: 1001–1007

Jacobs JJ, Keblusek P, Robanus-Maandag E, Kristel P, Lingbeek M, Nederlof PM, van Welsem T, van de Vijver MJ, Koh EY, Daley GQ and van Lohuizen M (2000) Senescence bypass screen identifies TBX2, which represses Cdkn2a (p19(ARF)) and is amplified in a subset of human breat cancers. *Nat Genet* **26**: 291–299

Jacobs JJL, Scheijen B, Voncken J-W, Kieboom K, Berns A and van Lohuizen M (1999) Bmi-1 collaborates with c-Myc in tumorigenesis by inhibiting c-Myc-induced apoptosis via INK4a/ARF. *Genes and Dev* **13**: 2678–2690

Jayaraman L and Prives C (1999) Covalent and noncovalent modifiers of the p53 protein. *Cell Mol Life Sci* **55**: 76–87

Jayaraman L, Murthy KGK, Zhu C, Curran T, Xanthoudakis S and Prives C (1997) Identification of redox/repair protein Ref-1 as a potent activator of p53. *Genes and Dev* **11**: 558–570

Jayaraman L, Moorthy NC, Murthy KG, Manley JL, Bustin M and Prives C (1998) High mobility group protein-1 (HMG-1) is a unique activator of p53. *Genes and Dev* **12**: 462–472

Jimenez GS, Nister M, Stommel JM, Beeche M, Barcarse EA, Zhang X-Q, O'Gorman S and Wahl G (2000) A transactivation-deficient mouse model provides insights into Trp53 regulation and function. *Nature Genet* **26**: 37–41

Joazeiro CAP and Weissman AW (2000) RING finger proteins: mediators of ubiquitin ligase activity. *Cell* **102**: 549–552

Jones SN, Roe AE, Donehower LA and Bradley A (1995) Rescue of embyonic lethality in Mdm2-deficient mice by absence of p53. *Nature* **378**: 206–208

Juan L-J, Shia W-J, Chen M-H, Yang W-M, Seto E, Lin Y-S and Wu C-W (2000) Histone deacetylases specifically down-regulate p53-dependent gene activation. *J Biol Chem* **275**: 20436–20443

Juven-Gershon T, Shifman O, Unger T, Elkeles A, Haupt Y and Oren M (1998) The Mdm2 oncoprotein interacts with the cell fate regulator Numb. *Mol Cell Biol* **18**: 3974–3982

Kamijo T, Zindy F, Roussel MF, Quelle DE, Downing JR, Ashmun RA, Grosveld G and Sherr CJ (1997) Tumor suppression at the mouse *INK4a* locus mediated by the alternative reading frame product p19$^{ARF}$. *Cell* **91**: 649–659

Kamijo T, Weber JD, Zambetti G, Zindy F, Roussel MF and Sherr CJ (1998) Functional and physical interactions of the ARF tumor suppressor with p53 and Mdm2. *Proc Natl Acad Sci USA* **95**: 8292–8297

Kapoor M and Lozano G (1998) Functional activation of p53 via phosphorylation following DNA damage by UV but not γ-irradiation. *Proc Natl Acad Sci USA* **95**: 2834–2837

Kastan MB, Zhan Q, El Deiry W-S, Carrier F, Jacks T, Walsh WV, Plunkett BS, Vogelstein B and Fornace Jr., A-J (1992) A mammalian cell cycle checkpoint pathway utilizing p53 and GADD45 is defective in ataxia-telangiectasia. *Cell* **71**: 587–597

Khosravi R, Maya R, Gottlieb T, Oren M, Shiloh Y and Shkedy D (1999) Rapid ATM-dependent phosphorylation of MDM2 precedes p53 accumulation in response to DMA damage. *Proc Natl Acad Sci USA* **96**: 14973–14977

Kirch HC, Flaswinkel S, Rumpf H, Brockmann D and Esche H (1999) Expression of human p53 requires synergistic activation of transcription from the p53 promoter by AP-1, NF-kappaB and Myc/Max. *Oncogene* **18**: 2728–2738

Kobet E, Zeng X, Zhu Y, Keller D and Lu H (2000) MDM2 inhibits p300-mediated p53 acetylation and activation by forming a ternary complex with the two proteins. *Proc Natl Acad Sci USA* **97**: 12547–12552

Komarov PG, Komarova EA, Kondratov RV, Christov-Tselkov K, Coon JS, Chernov MV and Gudkov AV (1999) A chemical inhibitor of p53 that protects mice from the side effects of cancer therapy. *Science* **285**: 1733–1737

Koumenis C, Alarcon R, Hammond E, Sutphin P, Hoffman W, Murphy M, Derr J, Taya Y, Lowe SW, Kastan M and Giaccia A (2001) Regulation of p53 by hypoxia; dissociation of transcriptional repression and apoptosis from p53-dependent transactivation. *Mol Cell Biol* **21**: 1297–1310

Kubbutat MHG and Vousden KH (1997) Proteolytic cleavage of human p53 by calpain: a potential regulator of protein stability. *Mol Cell Biol* **17**: 460–468

Kubbutat MHG, Jones SN and Vousden KH (1997) Regulation of p53 stability by Mdm2. *Nature* **387**: 299–303

Kubbutat MHG, Ludwig RL, Levine AJ and Vousden KH (1999) Analysis of the degradation function of Mdm2. *Cell Growth and Diff* **10**: 87–92

Lain S, Midgley C, Sparks A, Lane EB and Lane DP (1999) An inhibitor of nuclear export activates the p53 response and induces the localization of HDM2 and p53 to U1A-positive nuclear bodies associated with the PODS. *Exp Cell Res* **248**: 457–472

Lakin ND and Jackson SP (1999) Regulation of p53 in response to DNA damage. *Oncogene* **18**: 7644–7655

Li P-F, Dietz R and von Harsdorf R (1999) p53 regulates mitochondrial membrane potential through reactive oxygen species and induces cytochrome c-independent apoptosis blocked by Bcl-2. *EMBO J* **18**: 6027–6036

Lin Y and Benchimol S (1995) Cytokines inhibit p53-mediated apoptosis but not p53-mediated Gl arrest. *Mol Cell Biol* **15**: 6045–6054

Lin Y, Ma W and Benchimol S (2000) Pidd, a new death-domain-containing protein, is induced by p53 and promotes apoptosis. *Nature Genet* **26**: 124–127

Liu L, Scolnick DM, Trievel RC, Zhang HB, Marmorstein R, Halazonetis TD and Berger SL (1999) p53 sites acetylated in vitro by PCAF and p300 are acetylated in vivo in response to DNA damage. *Mol Cell Biol* **19**: 1202–1209

Ljungman M (2000) Dial 9-1-1 for p53: Mechanisms of p53 activation by cellular stress. *Neoplasia* **2**: 208–225

Lohrum M and Scheidtmann KH (1996) Differential effects of phosphorylation of rat p53 on transactivation of promoters derived from different p53 responsive genes. *Oncogene* **13**: 2527–2539

Lohrum MAE, Ashcroft M, Kubbutat MHG and Vousden KH (2000) Identification of a cryptic nucleolar localization signal in MDM2. *Nature Cell Biol* **2**: 179–181

Loughran O and La Thangue NB (2000) Apoptotic and growth-promoting activity of E2F modulated by MDM2. *Mol Cell Biol* **20**: 2186–2197

Lu W, Pochampally R, Chen L, Traidej M, Wang Y and Chen J (2000) Nuclear exclusion of p53 in a subset of tumors requires MDM2 function. *Oncogene* **19**: 232–240

Lundberg AS, Hahn WC, Gupta P and Weinberg RA (2000) Genes involved in senescence and immortalization. *Curr Opin Cell Biol* **12**: 705–709

Lundgren K, Montes de Oca Luna R, McNeill YB, Emerick EP, Spencer B, Barfield CR, Lozano G, Rosenberg MP and Finlay CA (1997) Targeted expression of MDM2 uncouples S phase from mitosis and inhibits mammary gland development independent of p53. *Genes and Dev* **11**: 714–725

Luo J, Chen D, Shiloh A and Gu W (2000) Deacetylation of p53 modulates its effect on cell growth and apoptosis. *Nature* **408**: 377–381

 *© 2001 Cancer Research Campaign*

Ma Y, Yuan R, Meng Q, Goldberg ID, Rosen EM and Fan S (2000) p53-independent down-regulation of Mdm2 in human cancer cells treated with adriamycin. *Mol Cell Biol Res Commun* **3**: 122–128

Maestro R, Dei Tos AP, Hamamori Y, Krasnokutsky S, Sartorelli V, Kedes L, Doglioni C, Beach DH and Hannon GJ (1999) *twist* is a potential oncogene that inhibits apoptosis. *Genes and Dev* **13**: 2207–2217

Maheswaran S, Englert C, Bennerr P, Heinrich G and Haber DA (1995) The WT1 gene product stabilizes p53 and inhibits p53-mediated apoptosis. *Genes and Dev* **9**: 2143–2156

Malkin D, Li FP, Strong LC, Fraumini Jr, JF, Nelson CE, Kim DH, Kassel J, Gryka MA, Bischoff FZ, Tainsky MA and Friend SH (1990) Germ line p53 mutations in a familial syndrome of breast cancer, sarcomas, and other neoplasias. *Science* **250**: 1233–1238

Marchenko ND, Zaika A and Moll UM (2000) Death signal-induced localization of p53 protein to the mitochondria. *J Biol Chem* **275**: 16202–16212

Marechal V, Elenbaas B, Piette J, Nicolas J-C and Levine AJ (1994) The ribosomal L5 protein is associated with mdm-2 and mdm-2-p53 complexes. *Mol Cell Biol* **14**: 7414–7420

Marin MC, Jost CA, Irwin MS, DeCaprio JA, Caput D and Kaelin Jr., WG (1998) Viral oncoproteins discriminate between p53 homolog p73. *Mol Cell Biol* **18**: 6316–6324

Maya R and Oren M (2000) Unmasking of phosphorylation-sensitive epitopes on p53 and Mdm2 by a simple western-phosphatase procedure. *Oncogene* **19**: 3213–3215

Mayo LD, Turchi JJ and Berberich SJ (1997) Mdm-2 phosphorylation by DNA-dependent protein kinase prevents interaction with p53. *Cancer Res* **57**: 5013–5016

McCormick F (2000) Interactions between adenovirus proteins and the p53 pathway: the development of ONYX-015. *Semin Cancer Biol* **10**: 453–459

McKay BC, Ljungman M and Rainbow AJ (1999) Potential roles of p53 in nucleotide excision repair. *Cancinog* **20**: 1389–1396

Meek DW (1999) Mechanisms of switching on p53: a role for covalent modifications. *Oncogene* **18**: 7666–7675

Meyn MS (1999) Ataxia-telangiectasia, cancer and the pathobiology of the ATM gene. *Clin Genet* **55**: 289–304

Midgley CA, Desterro JM, Saville MK, Howard S, Sparks A, Hay RT and Lane DP (2000) An N-terminal p14ARF peptide blocks Mdm2-dependent ubiquitination in vitro and can activate p53 in vivo. *Oncogene* **19**: 2312–2323

Miyashita T and Reed JC (1995) Tumor suppressor p53 is a direct transcriptional activator of the human bax gene. *Cell* **80**: 293–299

Momand J, Zambetti GP, George DL and Levine AJ (1992) The mdm-2 oncogene product forms a complex with the p53 protein and inhibits p53-mediated transactivation. *Cell* **69**: 1237–1245

Montes de Oca Luna R, Wagner DS and Lozano G (1995) Rescue of early embryonic lethality in *mdm2*-deficient mice by deletion of *p53*. *Nature* **378**: 203–206

Muller S, Berger M, Lehembre F, Seeler JS, Haupt Y and Dejean A (2000) c-Jun and p53 activity is modulated by SUMO-1 modification. *J Biol Chem* **275**: 13321–13329

Muslin AJ and Xing H (2000) 14-3-3 proteins: regulation of subcellular localization by molecular interference. *Cell Sig* **12**: 703–709

Nakano K and Vousden KH (2001) PUMA, a novel pro-apopototic gene, is induced by p53. *Mol Cell* **7**: 683–694

Nakano K, Balint E, Ashcroft M and Vousden KH (2000) A ribonucleotide reductase gene is a transcriptional target of p53 and p73. *Oncogene* **19**: 4283–4289

Nishimori H, Shiratsuchi T, Urano T, Kimura Y, Kiyono K, Tatsumi K, Yoshida S, Ono M, Kuwano M, Nakamura Y and Tokino T (1997) A novel brain-specific p53-target gene, BAI1, containing thrombospondin type 1 repeats inhibits experimental angiogenesis. *Oncogene* **15**: 2145–2150

O'Connor DJ and Lu X (2000) Stress signals induce transcriptionally inactive E2F-1 independently of p53 and Rb. *Oncogene* **19**: 2369–2376

Oda E, Ohki R, Murasawa H, Nemoto J, Shibue T, Yamashita T, Tokino T, Taniguchi T and Tanaka N (2000) Noxa, a BH3-only member of the Bcl-2 family and candidate mediator of p53-induced apoptosis. *Science* **288**: 1053–1058

Oda K, Arakawa H, Tanaka T, Matsuda K, Tanikawa C, Mori T, Nishimori H, Tamai K, Tokino T, Nakamura Y and Taya Y (2000) *p53AIP1*, a potential mediator of p53-dependent apoptosis, and its regulation by Ser-46-phosphorylated p53. *Cell* **102**: 849–862

Offer H, Zurer I, Banfalvi G, Reh'k M, Falcovitz A, Milyavsky M, Goldfinger N and Rotter V (2001) p53 modulates base excision repair activity in a cell cycle-specific manner after genotoxic stress. *Cancer Res* **61**: 88–96

Ohki R, Nemoto J, Mrasawa H, Oda E, Inazawa J, Tanaka N and Taniguchi T (2000) Reprimo, a new candidate mediator of the p53-mediated cell cycle arrest at the G2 phase. *J Biol Chem* **275**: 22627–22630

Oliner JD, Kinzler KW, Meltzer PS, George DL and Vogelstein B (1992) Amplification of a gene encoding a p53-associated protein in human sarcomas. *Nature* **358**: 80–83

Ongkeko WM, Wang XQ, Siu WY, Lau AWS, Yamashita K, Harris AL, Cox LS and Poon RYC (1999) MDM2 and MDMX bind and stabilize the p53-related protein p73. *Current Biology* **9**: 829–832

Owen-Schaub LB, Zhang W, Cusack JC, Angelo LS, Santee SM, Fujiwara T, Roth JA, Deisseroth AB, Zhang W-W, Kruzel E and Radinsky R (1995) Wild-type human p53 and a temperature sensitive mutant induce Fas/APO-1 expression. *Mol Cell Biol* **15**: 3032–3040

Pariat M, Carillo S, Molinari M, Salvat C, Debussche L, Bracco L, Milner J and Piechaczyk M (1997) Proteolysis by calpains: a possible contribution to degradation of p53. *Mol Cell Biol* **17**: 2806–2815

Pearson M, Carbone R, Sebastiani C, Cioce M, Fagioli M, Saito S, Higashimoto Y, Appella E, Minucci S, Pandolfi PP and Pelicci PG (2000) PML regulates p53 acetylation and premature senescence induced by oncogenic ras. *Nature* **406**: 207–210

Perry ME, Mendrysa SM, Saucedo LJ, Tannous P and Holubar M (2000) p76 (MDM2) inhibits the ability of p90 (MDM2) to destabilize p53. *J Biol Chem* **275**: 5733–5738

Phillips AC, Ernst MK, Bates S, Rice NR and Vousden KH (1999) E2F-1 potentiates cell death by blocking anti-apoptotic signaling pathways. *Mol Cell* **4**: 771–781

Pietenpol JA, Tokino T, Thiagaligam S, El-Deiry W, Kinzler KW and Vogelstein B (1994) Sequence-specific transcriptional activation is essential for growth suppression by p53. *Proc Natl Acad Sci USA* **91**: 1998–2002

Polyak K, Xia Y, Zweier JL, Kinzler KW and Vogelstein B (1997) A model for p53-induced apoptosis. *Nature* **389**: 300–305

Pomerantz J, Schreiber-Agus N, Liégeois NJ, Silverman A, Alland L, Chin L, Potes J, Chen K, Orlow I, Lee H-W, Cordon-Cardo C and DePinho RA (1998) The *Ink4a* tumor suppressor gene product, $p19^{Arf}$, interacts with MDM2 and neutralizes MDM2's inhibition of p53. *Cell* **92**: 713–723

Prabhu NS, Somasundaram K, Satyamoorthy K, Herlyn M and El-Deiry WS (1998) p73β, unlike p53, suppresses growth and induces apoptosis of human papillomavirus E6-expressing cancer cells. *Int J Oncol* **13**: 5–9

Prisco M, Hongo A, Rizzo MG, Sacchi A and Baserga R (1997) The insulin-like growth factor I receptor as a physiologically relevant target of p53 in apoptosis caused by interleukin-3 withdrawal. *Mol Cel Biol* **17**: 1084–1092

Radfar A, Unnikrishnan I, Lee HW, DePinho RA and Rosenberg N (1998) p19(Arf) induces p53-dependent apoptosis during abelson virus-mediated pre-B cell transformation. *Proc Natl Acad USA* **95**: 13194–13199

Rampino N, Yamamoto H, Ionov Y, Li Y, Sawai H, Reed JC and Perucho M (1997) Somatic frameshift mutations in the BAX gene in colon cancers of the microsatellite mutator phenotype. *Science* **275**: 967–969

Riemenschneider MJ, Buschges R, Wolter M, Reifenberger J, Bostrom J, Kraus JA, Schlegel U and Reifenberger G (1999) Amplification and overexpression of the MDM4 (MDMX) gene from 1q32 in a subset of malignant gliomas without TP53 mutation or MDM2 amplification. *Cancer Res* **59**: 6091–6096

Rodriguez MS, Desterro JMP, Lain S, Midgley CA, Lane DP and Hay RT (1999) SUMO-1 modification activates the transcriptional response of p53. *EMBO J* **18**: 6455–6461

Roth JA, Swisher SG and Meyn RE (1999) p53 tumor suppressor gene therapy for cancer. *Oncology* **13**: 148–154

Ryan KM and Vousden KH (1998) Characterization of structural p53 mutants which show selective defects in apoptosis, but not cell cycle arrest. *Mol Cell Biol* **18**: 3692–3698

Ryan KM, Ernst MK, Rice NR and Vousden KH (2000) Role of NF-kB in p53-mediated programmed cell death. *Nature* **404**: 892–897

Sakaguchi K, Sakamoto H, Lewis MS, Anderson CW, Erickson JW, Appella E and Xie D (1997) Phosphorylation of serine 392 stabilizes the tetramer formation of tumor suppressor protein p53. *Biochem* **36**: 10117–10124

Sakaguchi K, Herrera JE, Saito S, Miki T, Bustin M, Vassilev A, Anderson CW and Appella E (1998) DNA damage activates p53 through a phosphorylation-acetylation cascade. *Genes and Dev* **12**: 2831–2841

Sakaguchi K, Saito S, Higashimoto Y, Roy S, Anderson CW and Appella E (2000) Damage-mediated phosphorylation of human p53 threonine 18 through a cascade mediated by a caseine 1-like kinase. Effect on MDM2 binding. *J Biol Chem* **275**: 9278–9283

Scheffner M, Huibregtse JM, Viestra RD and Howley PM (1993) The HPV-16 E6 and E6-AP complex functions as a ubiquitin-protein ligase in the ubiquitination of p53. *Cell* **75**: 495–505

Selivanova G, Iotsova V, Okan I, Fritsche M, Ström M, Groner B, Grafström RC and Wiman KG (1997) Restoration of the growth suppression function of mutant *p53* by a synthetic peptide derived from the p53 C-terminal domain. *Nature Med* **3**: 632–638

© 2001 Cancer Research Campaign

Sengupta S, Vonesch J-L, Waltzinger C, Zheng H and Wasylyck B (2000) Negative cross-talk between p53 and the glucocorticoid receptor and its role in neuroblastoma cells. *EMBO J* **19**: 6051–6064

Sharp DA, Kratowicz SA, Sank MJ and George DL (1999) Stabilization of the MDM2 oncogene by interaction with the structurally related MDMX protein. *J Biol Chem* **274**: 38189–38196

Shaw P, Freeman J, Bovey R and Iggo R (1996) Regulation of specific DNA binding by p53: evidence for a role of O-glycosylation and charged residues at the carboxy-terminus. *Oncogene* **12**: 921–930

Sherr CJ and Weber JD (2000) The ARF/p53 pathway. *Curr Opin Genet Dev* **10**: 94–99

Shieh S-Y, Ikeda M, Taya Y and Prives C (1997) DNA damage-induced phosphorylation of p53 alleviates inhibition by MDM2. *Cell* **91**: 325–334

Shieh S-Y, Ahn J, Tamai K, Taya Y and Prives C (2000) The human homologs of checkpoint kinases Chk1 and Cds1 (Chk2) phosphorylate p53 at multiple DNA damage-inducible sites. *Genes and Dev* **14**: 289–300

Shvarts A, Steegenga WT, Riteco N, van Laar T, Dekker P, Bazuine M, van Ham RCA, van der Houven van Oordt W, Hateboer G, van der Ed AJ and Jochemsen AG (1996) MDMX: a novel p53-binding protein with some functional properties of MDM2. *EMBO J* **15**: 5349–5357

Sigalas I, Calvert AH, Anderson JJ, Neal DE and Lunec J (1996) Alternatively spliced *mdm2* transcripts with loss of p53 binding domain sequences: transforming ability and frequent detection in human cancer. *Nature Med* **2**: 912–917

Simbulan-Rosenthal CM, Rosenthal DS, Luo R and Smulson ME (1999) Poly(ADP-ribosyl)ation of p53 during apoptosis in human osteosarcoma cells. *Cancer Res* **59**: 190–194

Sionov RV, Moallem E, Berger M, Kazaz A, Gerlitz O, Ben-Neriah Y, Oren M and Haupt Y (1999) c-Abl neutralizes the inhibitory effect of Mdm2 on p53. *J Biol Chem* **274**: 8371–8374

Smith ML, Chen I-T, Zhan Q, Bae I, Chen C-Y, Gilmer TM, Kastan MB, O'Connor PM and Fornace AJ (1994) Interaction of the p53-regulated protein Gadd45 with proliferating cell nuclear antigen. *Science* **266**: 1376–1380

Smith ML, Ford JM, Hollander MC, Bortnick RA, Amundson SA, Seo YR, Deng CX, Hanawalt PC and Fornace AJ (2000) p53-mediated DNA repair responses to UV irradiation: studies of mouse cells lacking p53, p21 and/or gadd45. *Mol Cell Biol* **20**: 3705–3714

Soengas MS, Alarcon RM, Yoshida H, Giaccia AJ, Hakem R, Mak TW and Lowe SW (1999) Apaf-1 and caspase-9 in p53-dependent apoptosis and tumor inhibition. *Science* **284**: 156–159

Soengas MS, Capodieci P, Polsky D, Mora J, Esteller M, Opitz-Araya X, McCombie R, Herman JG, Gerald WL, Lazebnik YA, Cordón-Cardo C and Lowe SW (2001) Inactivation of the apoptosis effector *Apaf-1* in malignant melanoma. *Nature* **409**: 207–211

Srivistava S, Zou Z, Pirollo K, Blattner W and Chang EH (1990) Germ-line transmission of a mutated p53 gene in a cancer-prone family with Li-Fraumini syndrome. *Nature* **348**: 747–749

Stommel JM, Marchenko ND, Jimenez GS, Moll UM, Hope TJ and Wahl GM (1999) A leucine-rich nuclear export signal in the p53 tetramerization domain: regulation of subcellular localization and p53 activity by NES masking. *EMBO J* **18**: 1660–1672

Stott F, Bates SA, James M, McConnell BB, Starborg M, Brookes S, Palmero I, Hara E, Ryan KM, Vousden KH and Peters G (1998) The alternative product from the human *CDKN2A* locus, $p14^{ARF}$, participates in a regulatory feedback loop with p53 and MDM2. *EMBO J* **17**: 5001–5014

Takekawa M, Adachi M, Nakahata A, Nakayama I, Itoh F, Tsukuda H, Taya Y and Imai K (2000) p53-inducible Wip1 phosphotase mediated a negative feedback regulation of p38 MAPK-p53 signaling in response to UV radiation. *EMBO J* **23**: 6517–6526

Takimoto R and El-Deiry WS (2000) Wild-type p53 transactivates the KILLER/DR5 gene through an intronic sequence-specific DNA-binding site. *Oncogene* **19**: 1735–1743

Tanaka H, Arakawa H, Yamaguchi T, Shiraishi K, Fukuda S, Matsui K, Takei Y and Nakamura Y (2000) A ribonucleotide reductase gene involved in a p53-dependent cell-cycle checkpoint for DNA damage. *Nature* **404**: 42–49

Tanimura S, Ohtsuka S, Mitsui K, Shirouzu K, Yoshimura A and Ohtsubo M (1999) MDM2 interacts with MDMX through their RING finger domains. *FEBS Lett* **447**: 5–9

Tao W and Levine AJ (1999) Nucleocytoplasmic shuttling of oncoprotein Hdm2 is required for Hdm2-mediated degradation of p53. *Proc Natl Acad Sci USA* **96**: 3077–3080

Thornborrow EC and Manfredi JJ (1999) One mechanism for cell type-specific regulation of the bax promoter by the tumor suppressor p53 is dictated by the p53 response element. *J Biol Chem* **274**: 33747–33756

Unger T, Juven-Gershon T, Moallem E, Berger M, Vogt Sionov R, Lozano G, Oren M and Haupt Y (1999) Critical role for Ser20 of human p53 in the negative regulation of p53 by Mdm2. *EMBO J* **18**: 1805–1814

Van Antwerp DJ, Martin SJ, Kafri T, Green DR and Verma IM (1996) Suppression of TNFα induced apoptosis by NF-kB. *Science* **274**: 787–790

Vaziri H, West MD, Allsopp RC, Davison TS, Wu Y-S, Arrowsmith CH, Poirier GG and Benchimol S (1997) ATM-dependent telomere loss in aging human diploid fibroblasts and DNA damage lead to the post-translational activation of p53 protein involving poly(ADP-ribose) polymerase. *EMBO J* **16**: 6018–6033

Vogelstein B, Lane D and Levine AJ (2000) Surfing the p53 network. *Nature* **408**: 307–310

Vousden KH (2000) p53: death star. *Cell* **101**: 691–694

Wadgaonkar R and Collins T (1999) Murine double minute (MDM2) blocks p53-coactivator interaction, a new mechanisms for inhibition of p53-dependent gene expression. *J Biol Chem* **274**: 13760–13767

Waldman T, Kinzler KW and Vogelstein B (1995) p21 is necessary for the p53-mediated G1 arrest in human cancer cells. *Cancer Res* **55**: 5187–5190

Waldman T, Lengauer C, Kinzler KW and Vogelstein B (1996) Uncoupling of S phase and mitosis induced by anticancer agents in cells lacking p21. *Nature* **381**: 713–716

Wang X, Ohnishi K, Takahashi A and Ohnishi T (1998) Poly(ADP-ribosyl)ation is required for p53-dependent signal transduction induced by radiation. *Oncogene* **17**: 2819–2825

Wang XW, Zhan Q, Coursen JD, Khan MA, Kontny HU, Yu L, Hollander MC, O'Connor PM, Fornace AJJ and Harris CC (1999) GADD45 induction of a G2/M cell cycle checkpoint. *Proc Natl Acad Sci USA* **96**: 3706–3711

Wani MA, Zhu QZ, El-Mahdy M and Wani AA (1999) Influence of p53 tumor suppressor protein in bias of DNA repair and apoptotic response in human cells. *Carcinogenesis* **20**: 765–772

Waterman MJ, Stavridi ES, Waterman JL and Halazonetis TD (1998) ATM-dependent activation of p53 involves dephosphorylation and association with 14-3-3 proteins. *Nat Genet* **19**: 175–178

Weber JD, Taylor LJ, Roussel MF, Sherr CJ and Bar-Sagi D (1999) Nucleolar Arf sequesters Mdm2 and activates p53. *Nature Cell Biol* **1**: 20–26

Weber JD, Kuo M-L, Bothner B, DiGiammarino EL, Kriwacki RW, Roussel MF and Sherr CJ (2000) Cooperative signals governing the ARF-Mdm2 interaction and nucleolar localization of the complex. *Mol Cell Biol* **20**: 2517–2528

Wu G, Burns TF, McDonald ER, Jiang W, Meng R, Krantz ID, Kao G, Gan DD, Zhou JY, Muschel R, Hamilton SR, Spinner NB, Markowitz S, Wu G and El-Deiry WS (1997) KILLER/DR5 is a DNA damage-inducible p53-regulated death receptor gene. *Nat Genet* **17**: 141–143

Wu H and Lozano G (1994) NF-kappaB activation of p53. A potential mechanism for suppressing cell growth in response to stress. *J Biol Chem* **269**: 20067–20074

Wu L and Levine AJ (1997) Differential Regulation of the p21/WAF-1 and *mdm2* genes after high dose UV irradiation: p53-dependent and p53-independent regulation of the *mdm2* gene. *Mol Med* **3**: 441–451

Wu XW, Bayle JH, Olson D and Levine AJ (1993) The p53 mdm-2 autoregulatory feedback loop. *Genes and Dev* **7**: 1126–1132

Yam CH, Siu WY, Arooz T, Chiu CHS, Lau A, Wang XQ and Poon RYC (1999) MDM2 and MDMX inhibit the transcriptional activity of ectopically expressed SMAD proteins. *Cancer Res* **59**: 5075–5078

Yu J, Zhang L, Hwang PM, Kinzler KW and Vogelstein B (2001) PUMA induces the rapid apoptosis of colorectal cancer cells. *Mol Cell In press*

Yu ZK, Geyer RK and Maki CG (2000) MDM2-dependent ubiquitnation of nuclear and cytoplasmic p53. *Oncogene* **19**: 5892–5897

Zaika A, Marchenko N and Moll UM (1999) Cytoplasmically "sequestered" wild type p53 protein is resistant to Mdm2-mediated degradation. *J Biol Chem* **274**: 27474–27480

Zeimet AG, Riha K, Berger J, Widschwendter M, Hermann M, Daxenbichler G and Marth C (2000) New insights into p53 regulation and gene therapy for cancer. *Biochem Pharmacol* **60**: 1153–1163

Zeng X, Chen L, Jost CA, Maya R, Keller D, Wang X, Kaelin WGJ, Oren M, Chen J and Lu H (1999) MDM2 suppresses p73 function without promoting p73 degradation. *Mol Cell Biol* **19**: 3257–3266

Zeng X, Keller D, Wu L and Lu H (2000) UV but not gamma irradiation accelerates p53-induced apopotosis in teratocarcinoma cells by repressing MDM2 transcription. *Cancer Res* **60**: 6184–6188

Zhang W, Lu Q, Xie Z-J and Mellgren RL (1997) Inhibition of the growth of WI-38 fibroblasts by benzyloxycarbonyl-Leu-Leu-Try diazomethyl ketone: evidence that cleavage of p53 by a calpain-like protease is necessary for G1 to S-phase transition. *Oncogene* **14**: 255–263

 *© 2001 Cancer Research Campaign*

Zhang Y, Xiong Y and Yarbrough WG (1998) ARF promotes MDM2 degradation and stabilizes p53: *ARF-INK4a* locus deletion impairs both the Rb and p53 tumor suppression pathways. *Cell* **92**: 725–734

Zhou J, Ahn J, Wilson SH and Prives C (2001) A role for p53 in base excision repair. *EMBO J* **20**: 914–923

*© 2001 Cancer Research Campaign*
